# Demystifying the functions of the mitochondrial ORFan proteins in bivalves with doubly uniparental inheritance

**DOI:** 10.1098/rsbl.2024.0564

**Published:** 2025-01-15

**Authors:** Julie Brémaud, Alizée Debelli, Hajar Hosseini Khorami, Donald T. Stewart, Annie Angers, Bernard Angers, Sophie Breton

**Affiliations:** ^1^ Département de sciences biologiques, Université de Montréal, Montréal, QC, Canada; ^2^ Department of Biology, Acadia University, Wolfville, NS, Canada

**Keywords:** mitogenomics, mtDNA, ORFan genes, doubly uniparental inheritance, Bivalvia, sexual development

## Abstract

Strict maternal inheritance of mitochondria is known to be the rule in animals, but over 100 species across six orders of bivalves possess doubly uniparental inheritance (DUI) of mitochondria. Under DUI, two distinctive sex-specific mitogenomes coexist. In marine and freshwater mussels, each mitogenome has an additional protein-coding gene, called female- and male-specific open reading frame or *forf* and *morf*, respectively. The function(s) of the associated FORF and MORF proteins remain unknown. Herein, we show that these proteins present similar tissue expression patterns in two distantly related DUI species: MORF was only expressed in male gonads, whereas FORF was expressed in all tissues of both sexes in the marine mussel *Mytilus edulis* and the freshwater mussel *Venustaconcha ellipsiformis*. Moreover, MORF was only expressed during the reproductive season, while FORF presented no clear seasonality pattern in *M. edulis*. Immunocytochemistry revealed the presence of both proteins in mitochondria and acrosomes of late spermatids and mature sperm. We hypothesize that MORF has a key function in spermatogenesis, while FORF has a more general function in both sexes. We also propose that both proteins may be involved in the fertilization process. The involvement of MORF in paternal mitochondrial transmission is also discussed.

## 1. Introduction

Mitochondrial inheritance is known to be strictly maternal in metazoans, but some bivalves break the rule: they possess two distinct lineages of mitochondrial DNA that are inherited either maternally or paternally. Specifically, embryos receive mitochondria containing both types of mtDNA; however, embryos that will become females retain only the maternal mtDNA (referred to as the female or F mtDNA) and transmit it through to their female offspring. In contrast, embryos that will become males receive and retain both the paternal mtDNA (male or M mtDNA) as well as the F mtDNA but they only transmit the M mtDNA to their sons [1–5]. The F mtDNA is not passed on via the sperm [1–5]. In this system of doubly uniparental inheritance (DUI) of mitochondria, females are described as being typically homoplasmic for the F mtDNA and males as heteroplasmic for the F mtDNA, which predominates in somatic cells, and the M mtDNA, which dominates the male gonad tissue and sperm [1,6,7]. In the marine clam *Ruditapes philippinarum* (order Venerida), eggs and sperm are, respectively, homoplasmic for the F mtDNA and M mtDNA, but heteroplasmy has been detected at organelle level in undifferentiated germ cells of both sexes [8].

To date, DUI has been reported in >100 species from six bivalve orders [9], and as reported in the literature, the DNA divergence between the F and M mtDNAs that coexist in a single male can reach >40% in some species [10,11]. However, the ‘why and how’ of DUI remain unanswered. An interesting lead has emerged from the discovery of two mitochondrial ‘ORFans’, i.e. Open Reading Frames for proteins with no known homologues [12]: one in the F mtDNA (the *forf* gene coding for the female-specific open reading frame (FORF) protein) and one in the M mtDNA (*morf* coding for male-specific open reading frame (MORF) protein) [13,14]. The expression of FORF and MORF has been validated in the marine mussel *Mytilus edulis* (Bivalvia: Mytilida [15,16]) and the freshwater mussel *Venustaconcha ellipsiformis* (Bivalvia: Unionida [13,14]). The expression of MORF has also been validated in *R. philippinarum* [17,18]. The existence of these two sex-specific proteins suggests a sex-related function. In fact, several observations led to the hypothesis of a sex-determination or reproductive function, at least in freshwater mussels: (i) several independent transitions from gonochorism to hermaphroditism all marked by the loss of the M mtDNA and important alterations in the *forf* gene and (ii) the fact that no heteromorphic sex chromosomes have been found in any bivalve species [14,19]. It has also been proposed that key elements of the DUI system, such as FORF and MORF, might share similar functions in distantly related species [20]. However, their functions remain unknown.

Here, we intended to shed light on the biological functions of FORF and MORF in two phylogenetically distant DUI species, *M. edulis* and *V. ellipsiformis*. We first investigated the tissular expression of FORF and MORF and highlighted similarities between the two species but differences in the expression pattern of FORF and MORF. We also examined the seasonal expression of both proteins (i.e. before and during gametogenesis) as well as their subcellular localization in *M. edulis*. Globally, our results suggest functional similarities in *M. edulis* and *V. ellipsiformis* and potential novel roles for mitochondria-encoded proteins.

## 2. Material and methods

*Venustaconcha ellipsiformis* individuals were collected in July 2018, in the Root River (Minnesota, USA, 43.720444° N, −92.45941417535025° W) at the peak of their sexual maturity and shipped alive to the Université de Montréal (Montreal, Canada). Although *V. ellipsiformis* is state threatened, the specimens were collected under authority of the Minnesota DNR, and no permit was required (the sampled population was healthy at that time). Upon arrival in Montreal, specimens were all easily sexed by microscopic examination of gonad smears and dissected to obtain gonad, muscle, gill and foot tissues using precautions to avoid sample cross-contamination. All tissues were stored at −80°C until further use. *Mytilus edulis* individuals were purchased from fish markets in Montreal in 2022 and 2023 (January to May, and December). May is expected to be when they start breeding [21], but some individuals are reproductively mature in December and January [22]. All mussels came from the Prince Edward Island Aquafarm Inc. of New London (Canada), which utilizes native unispecific *M. edulis* seedstocks [23]. Individuals were sexed as above and sometimes validated by polymerase chain reaction (PCR) for some reproductively immature individuals (see below), and dissected to obtain gonad, muscle, gill and foot tissues. All samples were stored at −80°C.

When mature (or identifiable) gametes were not present, sex of *M. edulis* specimens was identified by PCR, which remains a highly reliable method for sexing *M. edulis* individuals according to a previous study [23]. This was done to validate our first evaluation by microscopy (i.e. after visualization of undifferentiated male or female germ cells). Total DNA was extracted from gonads using the Qiagen DNeasy Blood & Tissue kit (Qiagen). PCR amplification of sex-specific sequences was conducted using the primers MeMcytb_For (5′-TGAGGAGGGTGAACTGTGTG-3′) and MeMcox2_Rev (5′-TATTGGCAACCGGTTAATGC-3′), which amplify a 1370 pb product from the M mtDNA, and MeFcytb_For (5′-CCCTGTGGTAGGTGAAAGGA-3′) and MeFcox2_Rev (5′-CCTGGGGTTTACTCCACAAA-3′), which amplify a 1466 pb product from the F mtDNA. PCR was performed on a TProfessional Basic Thermocycler (Biometra) with the following profile: 2 min of denaturation at 95°C, followed by 35 cycles of 20 s at 95°C, 40 s of annealing at 58°C, 105 s of elongation at 72°C and a final elongation for 5 min at 72°C. PCR amplifications were carried out in 50 µl volumes comprising 5 µl of 10× Taq reaction buffer supplied by the manufacturer (final concentration 1×), 1 µl of dNTP mix (deoxynucleotide triphosphate, final concentration 200 µM), 2 µl of each primer (final concentration 0.4 µM), 0.25 µl of Taq DNA polymerase (final concentration 0.025 U µl^−1^; BioBasic), 1 μl of DNA extract (approx. 200 ng μl^−1^), and ddH_2_O up to 50 μl.

For western blotting, tissue samples (*n* = 3–5 for each tissue and sex or for each month and sex) were homogenized as in [16], and total protein concentration was estimated using the Bradford dosage method. Each homogenate was then mixed with LSB 5× loading buffer (glycerol 5.5 M, Tris–HCl 2 M, sodium dodecylsulfate 30%, dithiothreitol 1 M, β-mercaptoethanol 5% and 0.05% (w/v) bromophenol blue) and heated for 5 min at 95°C to denature proteins. Proteins were loaded (100 µg for *M. edulis* and 200 µg for *V. ellipsiformis*, an adjustment made due to difference in mitochondrial marker ATP5A band intensity) on tripartite SDS-PAGE tricine gels [24]. Electrophoresis was carried out overnight at room temperature (RT) at 45 V. Proteins were then transferred to a nitrocellulose membrane 0.45 µm, except for *V. ellipsiformis* FORF, for which polyvinylidene difluoride membranes (0.2 µm) produced better results because they are more efficient in binding smaller proteins. Ponceau R was used to reveal proteins on the membranes. Blocking was done by soaking the membranes in a BLOTTO buffer consisting of 5% non-fat instant skim milk powder diluted in phosphate buffered saline with Tween (monobasic sodium phosphate 50 mM, sodium chloride 150 mM, 0.05% Tween 20^©^ and pH 8) for 1 h at RT. Membranes were then incubated with primary anti-peptide antibodies (anti-FORF 1:1000 for *M. edulis*, anti-FORF 1:2000 for *V. ellipsiformis*, anti-MORF 1:2000 and anti-ATP5A (Abcam; ab14748) 1:4000 for both species) for 1.5 h at RT. Table 1 presents the custom rabbit anti-MORF and anti-FORF that were ordered from MédiMabs (Montreal, Canada) for *M. edulis* and from Life Technologies (New York, USA) for *V. ellipsiformis*. Membranes were washed with Tris-buffered saline (TBS; Tris-base 20 mM, sodium chloride 150 mM, 0.05% Tween 20^©^ and pH 7.6) three times for 10 min. Incubation with horeradish peroxidase-conjugated secondary antibodies (i.e. anti-rabbit for anti-MORFs and anti-FORFs and anti-mouse for anti-ATP-5A; both 1:2000) lasted 30 min. A second washing consisted of three 10 min rinses with TBS-T, followed by a final wash with TBS for 5 min. Immunoreactivity was recorded for all membranes by adding enhancer and substrate solutions (SuperSignal™ West Pico PLUS, Thermo Fisher Scientific), and protein signals were visualized on a FUSION FX system (Vilber Lourmat). The specificity of anti-FORF and anti-MORF antibodies was checked by adding the respective immunogenic synthetic peptide to each primary antibody solution before the incubation to competitively chelate every antigenic site and show attenuation of the specific bands.

**Table 1.** Antibodies produced against epitopes specific to MORF and FORF proteins of *M. edulis* and *V. ellipsiformis*. Amino acid sequences of FORF and MORF proteins are shown in the bottom four rows.

species	mtDNA	epitope	length of the reference protein (aa)	expected molecular weight (kDa)	references
*M. edulis*	F mtDNA	LISEASGAINAGKESFSDC	163	17	[15]
	M mtDNA	CFYTLNFPATACNSSSRL	94	11	[16]
*V. ellipsiformis*	F mtDNA	GSYPIKNSPASTNISDKT	89	10	this study
	M mtDNA	ESKKADKEIEGDDIKEKEK	299	35	this study
FORF *M. edulis* MSMLFGDSLLSVVDFSEVLCSWFKAGFLVKKDLLLSGVWDTFLSHKNSMFGMDAGDGGLCQGGEGDGAQVRVTPEAVWVGGDTAVNAGAEAAPDNAEGAGR YVGDGYALPLEEVGCSSVEESESAVAEPEVVSSGFEPVEQSGVLISEASGAINAGKESFSDC					
MORF *M. edulis* MSGLFKQQTNFSGESCKSFKNSNKATQKMWCVACVYKYTQKKKKKKKKYHKCLKMSCFYTLNFPATACNSSSRLCPPVFLYVKVYCWHMRELLY					
FORF *V. ellipsiformis* LVMKMKTQIMNLLNNKMVQKLIIIFTTGLFLMIILPSPFLLVSTKITYPELSLTDNPPEKNQPTSTSTASTGSYPIKNSPASTNISDKT					
MORF *V. ellipsiformis* MREVVNLKKLYFKTQHSLILRLISDLVSWLGFCLENYPILTLFMLFFTVLMFWGFVRGIVTLTEVFEEQQEKEVALGSLNKDKLEFEKNMGNLKMMEIELNKKMKAFELDKKVDRLKKEEFGLIKKVDTLKKEEFKFQGKLEELKAEVFELRKKVDKLKEEESMIEEKVDMMKMEWLSLDVKMNSLKKEEYESKKADKEIEGDDIKEKEKVFDIVDDEVGVEAKNIDKKKKLAKKKLGGVTKNSVKKKNTTIKHIIEGGDAITSAGNKAGVDIKKGIKKKYKKVIKKKPTEEGDGEVDK					

Immunocytochemistry was performed on *M. edulis* liberated sperm and eggs or cultured male mantle cells as in [16]. Cultured male mantle cells allow for the proliferation of male germ cells, especially spermatids [25]. The following primary antibody concentrations were used: anti-ATP-5A (1:300), anti-MORF (1:400) and anti-FORF (1:100). For secondary antibodies, Alexa Fluor 488-goat anti-rabbit green 1:400 was used for MORF and FORF and Alexa Fluor 594-goat anti-mouse red 1:400 for ATP5A. Immunoreactivity was visualized on an EVOS M5000 and a Leica Microsystems Mica.

## 3. Results

**Figure 1.**
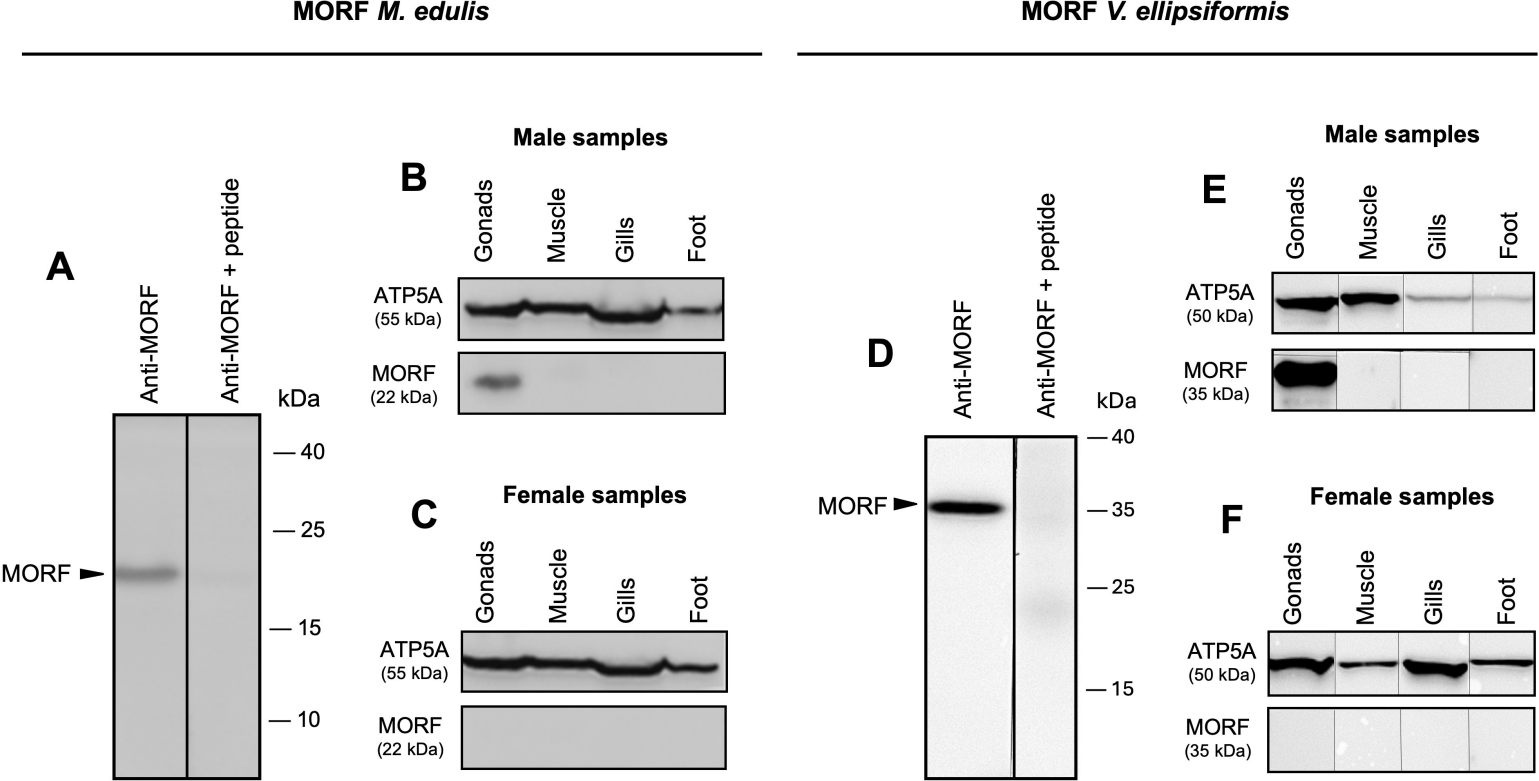
Western blotting of MORF in *Mytilus edulis* and *Venustaconcha ellipsiformis*. (A) Preadsorption experiment showing the attenuation of the MORF signal at proxy 22 kDa in *M. edulis* male gonads. (B) MORF immunoblotting in *M. edulis* male samples. (C) MORF immunoblotting in *M. edulis* female samples. (D) Preadsorption experiment showing the attenuation of the MORF signal at proxy 35 kDa in *V. ellipsiformis* male gonads. (E) MORF immunoblotting in *V. ellipsiformis* male samples. (F) MORF immunoblotting in *V. ellipsiformis* female samples.

**Figure 2.**
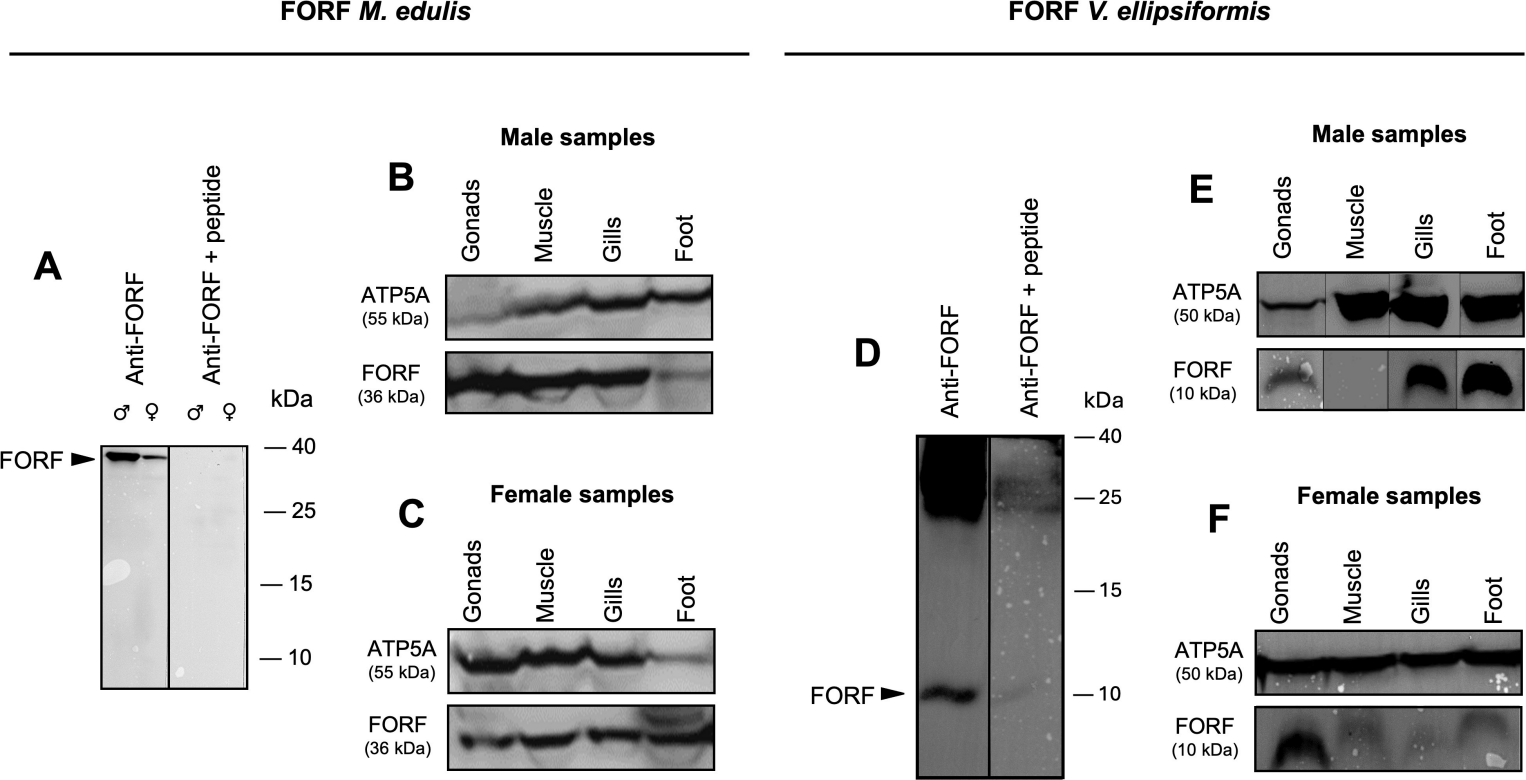
Western blotting of FORF in *Mytilus edulis* and *Venustaconcha ellipsiformis*. (A) Preadsorption experiment showing the attenuation of the FORF signal at proxy 36 kDa in *M. edulis* male and female gonads. (B) FORF immunoblotting in *M. edulis* male samples. (C) FORF immunoblotting in *M. edulis* female samples. (D) Preadsorption experiment showing the attenuation of the FORF signal at proxy 10 kDa in *V. ellipsiformis* female gonads. (E) FORF immunoblotting in *V. ellipsiformis* male samples. (F) FORF immunoblotting in *V. ellipsiformis* female samples.

Western blots revealed that MORF proteins are expressed in both *V. ellipsiformis* and *M. edulis* but only in male gonads (figure 1*a–f*; electronic supplementary material, figure S1*A*–*I*). The signal was of expected size in *V. ellipsiformis* (35 kDa; figure 1*d–f*) but higher than expected in *M. edulis* (22 kDa instead of 11 kDa; figure 1*a–c*), and the specificity of both anti-MORF antibodies was confirmed with preadsorption of the antigen (figure 1*a*,*d*). In contrast, FORF proteins were detected in all tissues in both species, apart from *V. ellipsiformis* male muscle samples (figure 2*a–f*; electronic supplementary material, figure S2*A*–*H*). Again, the signal was of expected size in *V. ellipsiformis* (10 kDa; figure 2*d–f*) but higher than expected in *M. edulis* (approx. 36 kDa instead of 17 kDa; figure 2*a–c*), and the specificity of both anti-FORF antibodies was also confirmed (figure 2*a*,*d*). The differences between the observed and expected molecular weight for FORF and MORF in *M. edulis* have already been discussed previously [15,16].

**Figure 3.**
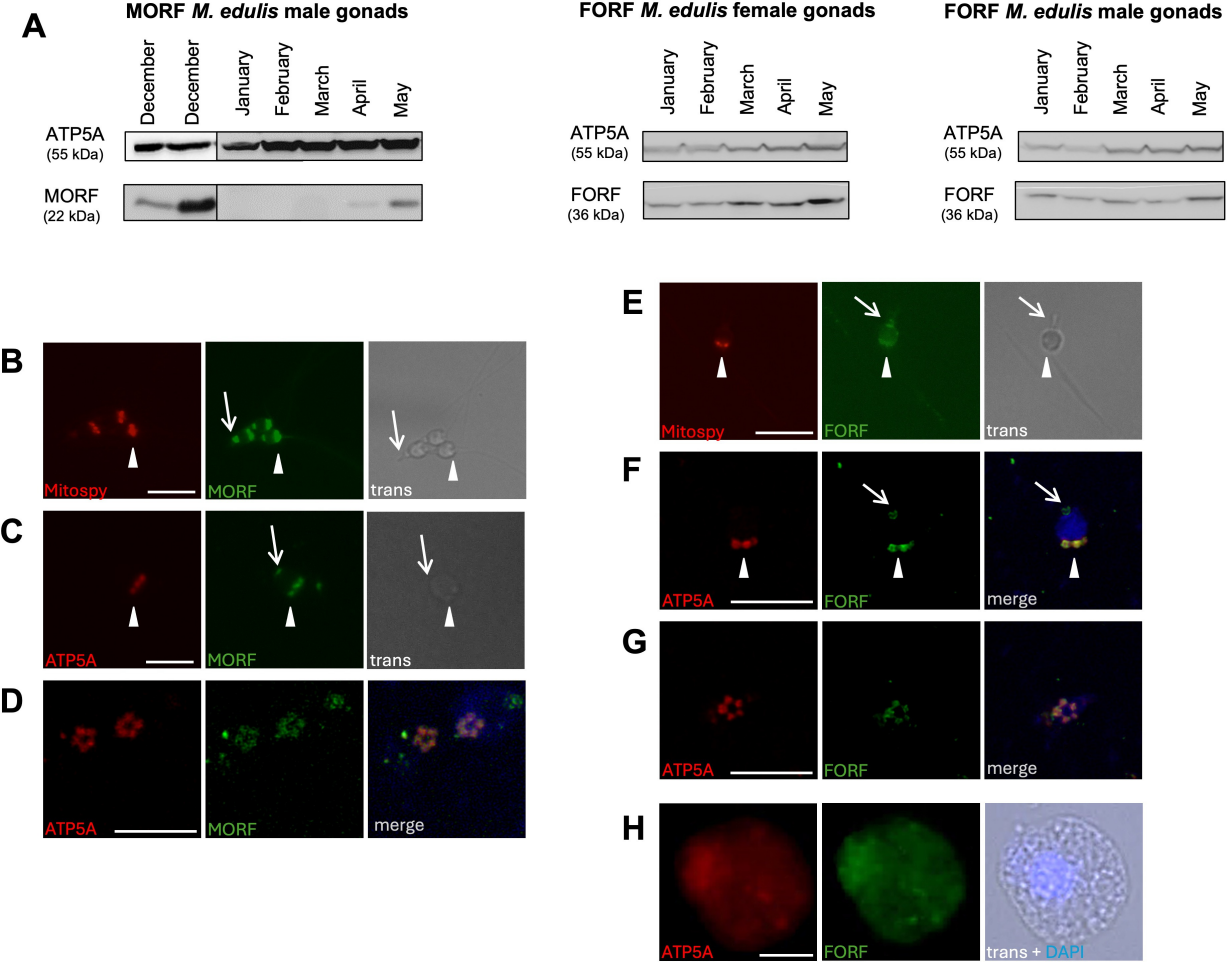
Temporal expression and subcellular localization of MORF and FORF in *Mytilus edulis*. (A) Western blotting showing the temporal expression patterns of MORF and FORF in *M. edulis* from January (no mature gametes) to May (mature gametes). MORF expression was also analysed by western blotting in some male individuals in December. (B–H) Immunocytochemistry performed on either liberated *M. edulis* sperm and eggs or cultured male mantle cells. (B) Sperm cell from male gonad tissue. MitoSpy Red colours mitochondria. MORF protein is in green. (C) Spermatids from cultured male mantle cells. MitoSpy Red colours mitochondria. MORF protein is in green. (D) Spermatids from cultured male mantle cells. Mitochondria are in red, MORF protein is in green and nucleus is in blue. (E) Sperm cell from male gonad tissue. MitoSpy Red colours mitochondria. FORF protein is in green. (F) Spermatids from cultured male mantle cells. Mitochondria are in red, FORF protein is in green and nucleus is in blue. (G) Spermatids from cultured male mantle cells. Mitochondria are in red, FORF protein is in green and nucleus is in blue. (H) Immature oocyte. Mitochondria are in red, FORF protein is in green and nucleus is in blue. Big arrowhead indicates mitochondria, small arrow indicates acrosome. Scale bars, 10 µm.

To further explore the functional aspects of MORF and FORF, we investigated their seasonal expression in *M. edulis*, which generally start breeding in May [21]. No expression of MORF was observed in male gonads for the first three months of the year for all samples tested (even though the M mtDNA was well detected in all samples), while MORF was well expressed in all male gonads tested at the beginning of the breeding season in May (figure 3*a*; electronic supplementary material, figure S3*A*,*B*). MORF expression was also observed in some male individuals with mature and motile gametes in December. In contrast, FORF was detected in all first five months of the year in both female and male gonads (figure 3*a*; electronic supplementary material, figure S3*C*).

Immunocytochemistry was also performed on either liberated sperm and eggs (figure 3*b*,*e*,*h*; electronic supplementary material, figure S3*D*) or cultured male mantle cells (figure 3*c*,*d*,*f*,*g*; electronic supplementary material, figure S3*D*), which allow for the proliferation and observation of male germ cells, especially spermatids [25]. The results indicated that MORF co-localized with the mitochondrial markers ATP5A or MitoSpy Red in spermatids and mature sperm (figure 3*b*,*c*), and MORF and a slight MitoSpy Red signal were also detected in the sperm acrosome (figure 3*b*). FORF co-localized with MitoSpy Red in eggs (figure 3*h*), late spermatids (figure 3*f*) and mature sperm (figure 3*e*). FORF was also detected in the acrosome (figure 3*e*,*f*).

## 4. Discussion

Herein, we present evidence suggesting that the sex-specific mitochondrial ORFan proteins in bivalves with DUI share similar functions in phylogenetically distant species. Our results showed, in the marine mussel *M. edulis* and the freshwater mussel *V. ellipsiformis*, that the MORF protein is exclusively expressed in male gonads, whereas the FORF protein is expressed in all tissues of both sexes (except in male muscles in *V. ellipsiformis*). Our results also showed, in *M. edulis*, that MORF is only expressed during the reproductive season, while FORF is expressed throughout the year. Moreover, immunocytochemistry revealed the presence of both proteins in mitochondria and acrosomes of late spermatids and mature sperm.

The detection of MORF exclusively in male gonads is particularly interesting, knowing that the presence and expression (transcription) of the M mtDNA in somatic tissues of males (often) and females (occasional) have been reported in both *M. edulis* and *V. ellipsiformis* [26,27]. This suggests that MORF may be translated at very low levels in somatic tissues (i.e. undetectable by western blot) or that silencing mechanisms exist for MORF at the post-transcriptional, translational and/or post-translational levels. MORF also seems to be seasonally expressed in testis, i.e. at the beginning of (and during) the reproductive season when male gametes are mostly mature and motile. For example, it was not detected in any *M. edulis* male sampled in February and March (with very few and immotile sperm) but was detected in all males sampled in May and December (with many motile sperm). It is not unusual to observe more than one spawning event in *Mytilus* species, and reproductively mature individuals are often observed in the winter [21,22]. MORF expression was also observed in late spermatids, i.e. the final stage of spermatogenesis eventually leading to mature sperm (see below). Also, it must be noted that the expression of MORF did not seem to depend on sperm quantity since high densities of mature sperm (but mostly non-motile) were also found in male individuals from April (A. Debelli 2022, personal observation). A similar seasonal protein expression pattern independent of sperm density has been reported for another M mtDNA-encoded protein (MCOX2) in *V. ellipsiformis*, although MCOX2 also showed a low uniform expression in male somatic tissues in this species [28].

Globally, the disparate tissular and temporal expression patterns of MORF and FORF indicate that the two proteins have distinct biological functions. While FORF appears to have a more general function as it is expressed in all tissues of both sexes (except male muscles in *V. ellipsiformis*), the exclusive presence of MORF in male gonads during the reproductive season suggests a possible role in spermatogenesis, ensuring the formation of mature sperm. This hypothesis is supported by previous studies on ‘role-reversal events’ in *Mytilus* species [19]. Specifically, intermolecular recombination events between F and M mitogenomes have previously been reported in *Mytilus* spp., which resulted in F mtDNAs that become transmitted through sperm. These role-reversed mtDNAs are mostly F mitogenomes but with M control region segments containing the *morf* gene, which suggests that an intact MORF protein might be necessary to produce sperm [19].

At the subcellular level, both proteins co-localized with mitochondrial markers in *M. edulis* gametes: MORF was detected in sperm mitochondria and FORF in eggs and sperm mitochondria. In *M. edulis*, sperm usually possess five big mitochondria in a star-shaped formation [29]. It is from the mid- to the late-spermatid stage that mitochondria are reduced to five in number, assembled to form a ‘star or rosette’ and restricted in position to one juxtanuclear region which will eventually become the sperm middle piece [29]. The presence of MORF and FORF in sperm mitochondria was somewhat expected since both proteins were previously detected in sperm mitochondria of both *M. edulis* and *V. ellipsiformis* [15,30]. The presence of FORF in *M. edulis* egg mitochondria was also expected, and since previous studies in *V. ellipsiformis* showed that FORF is also present outside egg mitochondria (i.e. in cytoplasm and nucleoplasm) indicating nonoxidative phosphorylation functions [14], we expect similar results in *M. edulis* in future studies. Finally, we also expected to detect both MORF and FORF in late spermatid mitochondria since undifferentiated germ cells supposedly contain F and M mtDNA-encoded proteins [8]. This was reported in *R. philippinarum* but remains to be demonstrated in other DUI species. Because mature sperm are apparently homoplasmic for the M mtDNA, it is still unclear whether FORF is a long-lived mitochondrial protein that survives throughout the spermatogenesis or if it exported from ‘female mitochondria’ and imported in sperm mitochondria by still unknown mechanisms [15]. Moreover, because our anti-FORF and anti-MORF antibodies were both produced by rabbits, we were not able to verify whether the two proteins were present in the mitochondria of the same or in different spermatids, although the spermatids we observed all appeared to express FORF or MORF, suggesting that they all express both proteins (electronic supplementary material, figure S4).

A result that was rather unexpected was the presence of both MORF and FORF in the acrosome. In *M. edulis* late spermatids, the acrosome is rounded-shaped before extending to form the mature sperm acrosomal vesicle [29], and this is indeed what we observed in our immunofluorescence results. A similar signal was also visible for FORF in a previous study, but not discussed [15]. The presence of MORF in the sperm acrosome was already reported by Debelli *et al.* [16], but the present study describes for the first time the presence of FORF in the sperm acrosome. Again, we were not able to verify whether the two proteins were found in the same acrosome or not. Nevertheless, our results raise the questions of whether the two proteins are both necessary for acrosome formation, and if they are involved in fertilization. Interestingly, the sperm acrosome in mice has been shown to be formed by condensed germ cell mitochondria [31,32], and this could be the same in *M. edulis*, as we detected an ATP5/MitoSpy signal in the acrosome. Ren *et al*. [31,32] also showed that the proteome of the acrosome in mice contains mitochondrial proteins, and Li *et al.* [33] recently demonstrated that mitochondrial proteins are involved in acrosome formation in the Chinese mitten crab *Eriocheir sinensis*. Again, a similar process could be occurring in *Mytilus* spp. Furthermore, sperm acrosomal proteins in *Mytilus* spp. include gamete recognition proteins [34], suggesting that MORF and FORF could also be involved in gamete–gamete interaction. It is worth noting that a nuclear-encoded mitochondrial protein in *Arabidopsis thaliana* is known to be essential for fertilization, specifically for gamete recognition [35]. Although further work will be necessary to demonstrate whether MORF and FORF are indeed implicated in acrosome formation during spermatogenesis and/or in gamete recognition, our results are in line with recent studies showing that mitochondria-derived elements (i.e. small non-coding RNAs of mitochondrial origin) may be involved in sexual development (germ line formation) in bivalves with DUI [36,37].

Finally, our confocal microscopy results showed clear superposition of FORF or MORF and the mitochondrial marker ATP5 in late spermatids. MORF also seemed to form a sort of intra- and inter-mitochondrial network in/around spermatid mitochondria, although electron microscopy will be needed to confirm that MORF (or FORF) is found at the mitochondria outer membrane. It is tempting to speculate that MORF could be involved in the establishment of the stable ‘five-mitochondria rosette’ structure during spermatogenesis. A similar mechanism has been reported in mice, where a mitochondrial surface protein specific to germ cells was shown to be essential for proper spermatogenesis by clustering mitochondria through self-interaction [38]. In *M. edulis*, the ‘mitochondria rosette’ structure may also be essential to ensure the transmission of sperm mitochondria to the egg and their aggregation in the blastomere that will give rise to the gonad in male embryos [4]. However, the relationship between MORF (and maybe FORF) and mitochondria star-shaped formation in *M. edulis* sperm as well as its impact on spermatogenesis and paternal mitochondrial transmission remains unclear and warrants further investigations.

## Data Availability

All data are available online as electronic supplementary material [39].
